# DNA methylation and gene expression profiles to identify childhood atopic asthma associated genes

**DOI:** 10.1186/s12890-021-01655-8

**Published:** 2021-09-15

**Authors:** Rui Chen, Li-Zhen Piao, Ling Liu, Xiao-Fei Zhang

**Affiliations:** Department of Pediatrics, The Third Hospital of Jilin University, Changchun, 130033 Jilin China

**Keywords:** Atopic asthma, Gene expression profile, DNA methylation profile, miRNA

## Abstract

**Background:**

Asthma is a chronic inflammatory disorder of the airways involving many different factors. This study aimed to screen for the critical genes using DNA methylation/CpGs and miRNAs involved in childhood atopic asthma.

**Methods:**

DNA methylation and gene expression data (Access Numbers GSE40732 and GSE40576) were downloaded from the Gene Expression Omnibus database. Each set contains 194 peripheral blood mononuclear cell (PBMC) samples of 97 children with atopic asthma and 97 control children. Differentially expressed genes (DEGs) with DNA methylation changes were identified. Pearson correlation analysis was used to select genes with an opposite direction of expression and differences in methylation levels, and then Gene Ontology (GO) function and Kyoto Encyclopedia of Genes and Genomes (KEGG) pathway analysis were performed. Protein–protein interaction network and miRNA–target gene regulatory networks were then constructed. Finally, important genes related to asthma were screened.

**Results:**

A total of 130 critical DEGs with DNA methylation changes were screened from children with atopic asthma and compared with control samples from healthy children. GO and KEGG pathway enrichment analysis found that critical genes were primarily related to 24 GO terms and 10 KEGG pathways. In the miRNA–target gene regulatory networks, 9 KEGG pathways were identified. Analysis of the miRNA–target gene network noted an overlapping KEGG signaling pathway, hsa04060: cytokine-cytokine receptor interaction, in which the gene *CCL2*, directly related to asthma, was involved. This gene is targeted by eight asthma related miRNAs (*hsa-miR-206*, *hsa-miR-19a*, *hsa-miR-9*,*hsa-miR-22*, *hsa-miR-33b*, *hsa-miR-122*, *hsa-miR-1*, and *hsa-miR-23b*). The genes *IL2RG* and *CCl4* were also involved in this pathway.

**Conclusions:**

The present study provides a novel insight into the underlying molecular mechanism of childhood atopic asthma.

## Background

Asthma is a respiratory disease caused by the interaction of genetic and environmental factors, known to be mediated by epigenetics [1]. Approximately 334 million people worldwide suffer from asthma. Childhood asthma mortality varies from 0.0 to 0.7 per 100,000 [2]. Candidate genes for asthma are wide-spread throughout the genome. There are multiple genes involved which may affect expression of the asthma phenotype. Different genes are related to childhood and adult asthma resulting in a different physiological foundation, and different methods of treatment for the two diseases [3, 4].

Genetic variants drive the onset and development of asthma, including cytotoxic T-lymphocyte-associated protein 4 (*CTLA4*) and interleukin-10 (*IL-10*) which are involved in immune system regulation and inflammation [5, 6]. DNA methylation, such as of the gene encoding the β-2 adrenergic receptor, is the most common epigenetic mechanism in the pathogenesis of asthma, and can change gene expression in asthmatic patients [7–9]. Acevedo et al. reported that regional DNA methylation and mRNA levels at the Gasdermin B/ORMDL sphingolipid biosynthesis regulator 3 locus were associated with the risk of childhood asthma [10]. Nicodemus-Johnson et al. reported that the type 2 cytokine IL-13 is a key mediator, and it is upregulated in asthma [11]. They found that a single exposure of IL-13 may induce DNA methylation changes in an asthmatic’s airway cells and contribute to various asthma phenotypes. Brand et al. found that the epigenetic regulation of T cells can influence the sensitization and progress of experimental asthma [12]. Based on these findings, it is expected that biomarkers for the early diagnosis of asthma can be determined from the level of gene expression and methylation regulation.

In a prior study, Yang et al. demonstrated that DNA methylation at specific gene loci are associated with asthma based on GSE40736 data set, and suggested that epigenetic changes might play a role in establishing the immune phenotype associated with asthma. However, the results of that analysis were only at the DNA level [13]. In the current study, we aimed to identify critical genes and miRNAs in the progression of childhood atopic asthma. We downloaded DNA methylation and gene expression data from the GEO database, and screened critical differentially expressed genes (DEGs) with significant methylation changes from samples obtained from atopic asthmatic patients and compared them with samples from healthy controls. Then we identified the Gene Ontology (GO) function and Kyoto Encyclopedia of Genes and Genomes (KEGG) pathways of these DEGs and constructed a gene co-expression and miRNA–target gene regulatory network. The aim of this study is to potentially provide novel diagnostic biomarkers in the nasal epithelia of children with atopic asthma.

## Methods

### DNA methylation and gene expression data resource

The DNA methylation dataset, superserie GSE40736, was downloaded from the National Center of Biotechnology Information Gene Expression Omnibus (GEO) database(https://www.ncbi.nlm.nih.gov/) [14], which contained two subseries (GSE40732 and GSE40576) that are gene expression and methylation level detection spectra, respectively. Each set contains 194 peripheral blood mononuclear cell (PBMC) samples of 97 children with atopic asthma and 97 control children. The GSE40732 data set was tested on the platform of Nimble Gen Homo sapiens Expression Array. The GSE40576 data set was tested on the Illumina HumanMethylation450 BeadChip platform.

### Data preprocessing and differentially expressed gene screening

After downloading the original microarray data, the limma package in R software (version3.1.3, https://bioconductor.org/packages/release/bioc/html/limma.html) [15] was used to normalize the DNA methylation and gene expression data. DNA hypomethylation and hypermethylation were common cancer hallmarks. False discovery rate (FDR) values and fold change values were calculated by using the limma package to evaluate the DEGs and differentially methylated genes (DMGs) between the disease and control groups. An FDR < 0.05 and |log2FC|> 0.5 were considered to be threshold values.

Moreover, the pheatmap package (Version 1.0.8, https://cran.r-project.org/package=pheatmap) [16] in R software was used to perform the bidirectional hierarchical clustering analysis for the gene expression and methylation values based on Euclidean distance [17, 18]. The pheatmap was constructed to visualize gene expression values.

### Gene ontology function and KEGG pathway analysis for DEGs and DMGs

Initially, we compared the collection of DEGs and DMGs, kept the intersection of the two data sets, and analyzed the overall correlation between the degree of difference in methylation and expression levels. The cor.test function (https://stat.ethz.ch/R-manual/R-devel/library/stats/html/cor.test.html) was used to calculate the Pearson correlation coefficient. The expression and methylation level differences in opposite directions were reserved for further analysis. Subsequently, the Database for Annotation, Visualization and Integrated Discovery tool, version 6.8 [19, 20] (DAVID, https://david.ncifcrf.gov/) was used to perform GO function and KEGG pathway enrichment analysis for the mRNAs with an opposite direction of difference in expression and methylation levels. The threshold value was considered to be *P* < 0.05.

### Analysis of protein–protein interaction network

String, version 10.5 [21] (https://string-db.org/), was used to search for the interaction between gene product proteins for genes with opposite expression and methylation levels, and an interactive network was built. The interactive network was visualized by Cytoscape version 3.7.2 software [22] (http://www.cytoscape.org/). The GO biological process and KEGG signal pathway analysis based on DAVID were then performed on the gene nodes that constituted the interaction network. The threshold value was considered to be *P* < 0.05.

### MiRNA–target gene regulatory network construction

We used the Human MicroRNA Disease Database [23] (HMDD, http://www.cuilab.cn/hmdd) to search for miRNAs directly associated with asthma. The target genes of asthma miRNAs were then screened using the starBase version 2.0 database [24] (http://starbase.sysu.edu.cn/). The starBase database provides the comprehensive target gene prediction information from five databases: TargetScan, picTar, RNA22, PITA, and miRanda. We selected regulatory relationships included in at least one of the databases as miRNA-target gene relationship pairs to construct a miRNA -mRNA regulation relationship. Cytoscape 3.7.2 was used to display the networks. Finally, KEGG pathway analysis for target genes was performed using DAVID software.

### Selection and mechanism analysis of candidate agents

In the Comparative Toxicogenomics Database, 2019 update [25] (http://ctd.mdibl.org/), using “asthmatic” as a keyword, we searched for KEGG pathways and genes directly related to asthma, and compared them with pathways in which the genes in the constructed interaction network were significantly involved in the relevant pathways. We selected disease pathways with direct involvement of the asthma genes, constructed this part of the network separately, screened genes directly related to the disease, and conducted mechanism research through the important pathways of gene participation.

## Results

### Differentially expressed genes and methylated sites screening

**Fig. 1:**
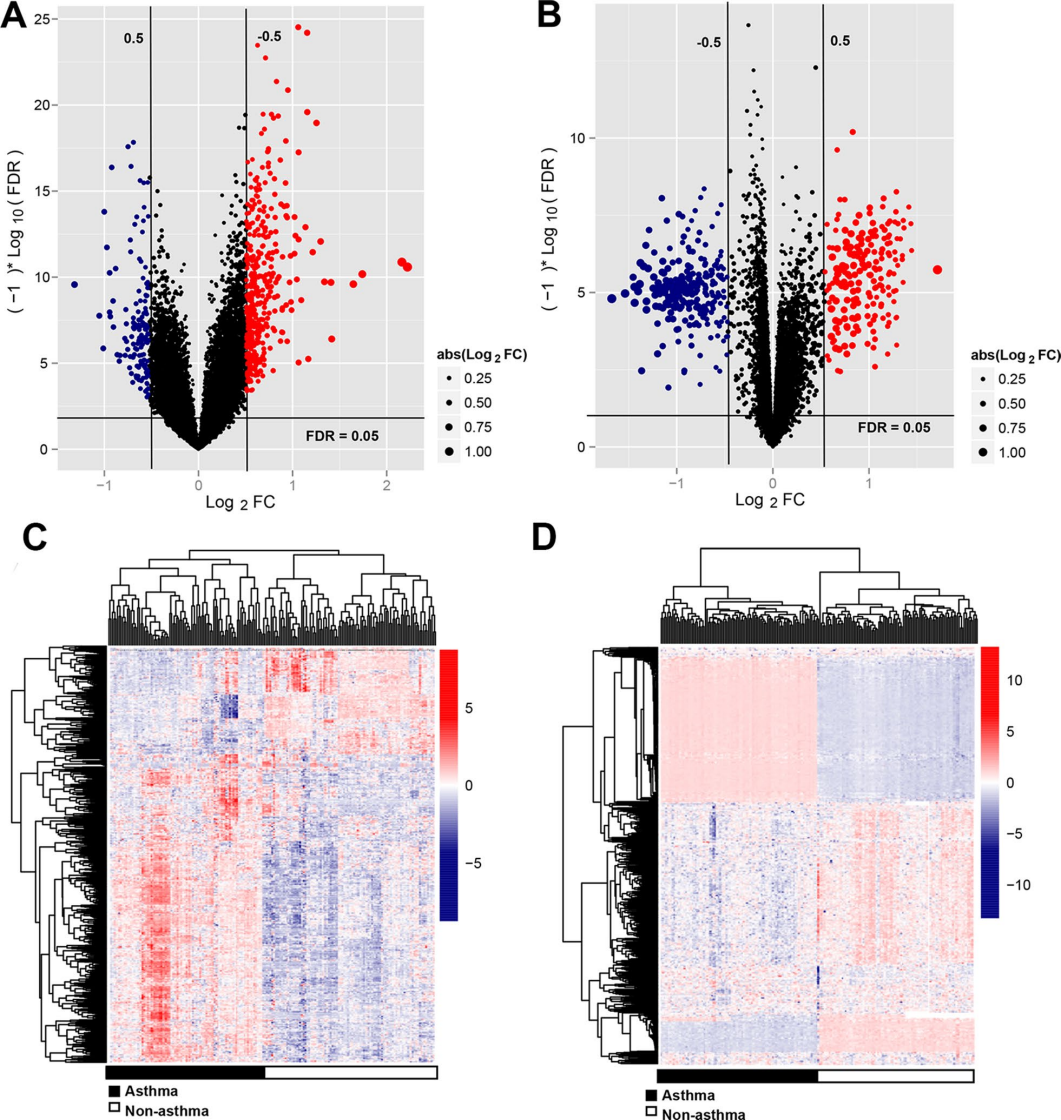
Volcano plot for the differentially expressed genes (**a**) and DNA methylation data (**b**). The horizontal dotted line represents an FDR = 0.05 threshold line;the red vertical dotted line represents the | logFC | > 0.5 threshold line; the red and blue dots represent significantly up-regulated and down-regulated DEGs and DMGs, respectively; and the black dots represent the non-differential expression genes. Bidirectional hierarchical clustering heatmaps for differentially expressed genes (**c**) and differentially expressed methylation regions (**d**). Black represents asthmatic samples and white represents healthy controls. FDR, false discovery rate; FC, fold change; DEGs, differentially expressed genes; DMGs, differentially methylated genes

Expression and methylation level files were downloaded. A total of 933 (239 downregulated and 694 upregulated) DEGs and 751 (412 hypomethylated and 339 hypermethylated) DMGs were identified between the asthmatic and healthy control groups. Volcano plots for the DEGs and differentially methylated sites were shown in Fig. 1a and b. After screening DEGs and DMGs from the gene expression and methylation profiles, the corresponding gene expression and signal values were visualized in bidirectional hierarchical clustering heatmaps (Fig. 1c, d). As can be seen in the figure, the difference between the selected DEGs and DMGs of the asthma and control groups is significant. The bidirectional hierarchical cluster heatmap revealed that the samples were clearly divided into two groups based on the screened DEGs and DMGs.

### Gene ontology and KEGG pathway analysis

**Fig. 2:**
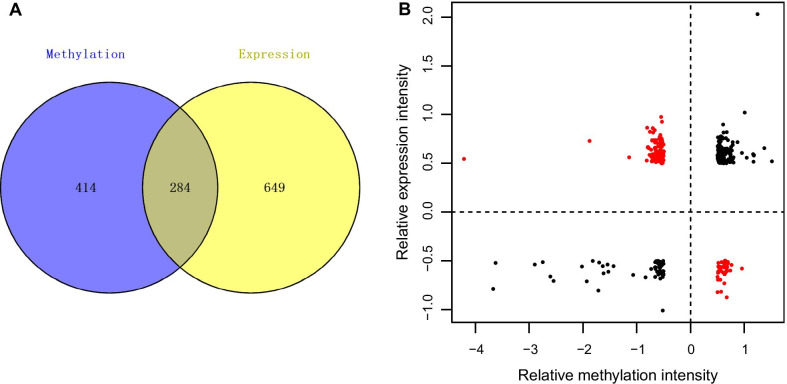
Comparison of DMGs and DEGs in a set of venn diagrams (**a**). Distribution map for differentially expressed genes with DNA methylation changes (**b**). The horizontal axis represents the degree of significant methylation difference, and the vertical axis represents the degree of gene expression difference. Red dots represent genes with opposite degrees of expression and methylation levels. DEGs, differentially expressed genes; DMGs, differentially methylated genes

We screened a total of 284 intersection genes that were differentially expressed in the DNA methylation and gene expression data set, and analyzed the relationship between gene expression and DNA methylation changes by calculating the correlation coefficient (Fig. 2a). Critical gene expression levels and the DNA methylome are shown in Fig. 2b. We reserved 130 genes for further analysis whose expression and methylation levels differed in opposite levels. Among these, there were 35 genes with hypermethylation and decreased expression and 95 genes with hypomethylation and increased expression. GO and KEGG pathway enrichment analyses showed that the critical genes were primarily related to 24 GO terms and 10 pathways (Table 1). The GO identified genes were involved in cellular functions including cellular defense response and oxidation reduction. The gene pathways were involved in multiple areas including natural killer cell mediated cytotoxicity, valine, leucine and isoleucine degradation, and steroid hormone biosynthesis.

**Table 1:** GO BPs and KEGG pathways significantly related to 130 differential genes

Category	Term	Count	PValue
Biology process	GO:0006968 ~ cellular defense response	5	1.06E−03
	GO:0006952 ~ defense response	13	1.90E−03
	GO:0055114 ~ oxidation reduction	12	7.47E−03
	GO:0051494 ~ negative regulation of cytoskeleton organization	4	7.79E−03
	GO:0002696 ~ positive regulation of leukocyte activation	5	7.86E−03
	GO:0050867 ~ positive regulation of cell activation	5	9.21E−03
	GO:0002703 ~ regulation of leukocyte mediated immunity	4	1.03E−02
	GO:0019835 ~ cytolysis	3	1.04E−02
	GO:0051129 ~ negative regulation of cellular component organization	5	2.10E−02
	GO:0006922 ~ cleavage of lamin	2	2.20E−02
	GO:0006923 ~ cleavage of cytoskeletal proteins during apoptosis	2	2.20E−02
	GO:0010639 ~ negative regulation of organelle organization	4	2.28E−02
	GO:0006865 ~ amino acid transport	4	2.90E−02
	GO:0042330 ~ taxis	5	3.08E−02
	GO:0006935 ~ chemotaxis	5	3.08E−02
	GO:0016310 ~ phosphorylation	12	3.39E−02
	GO:0002694 ~ regulation of leukocyte activation	5	3.46E−02
	GO:0051251 ~ positive regulation of lymphocyte activation	4	3.51E−02
	GO:0001562 ~ response to protozoan	2	3.64E−02
	GO:0043242 ~ negative regulation of protein complex disassembly	3	3.68E−02
	GO:0002697 ~ regulation of immune effector process	4	3.88E−02
	GO:0050865 ~ regulation of cell activation	5	4.07E−02
	GO:0001817 ~ regulation of cytokine production	5	4.52E−02
	GO:0006690 ~ icosanoid metabolic process	3	4.72E−02
KEGG pathway	hsa04650:Natural killer cell mediated cytotoxicity	5	3.59E−03
	hsa00280:Valine, leucine and isoleucine degradation	3	6.42E−03
	hsa00140:Steroid hormone biosynthesis	3	6.95E−03
	hsa04080:Neuroactive ligand-receptor interaction	6	9.17E−03
	hsa00590:Arachidonic acid metabolism	3	9.75E−03
	hsa04514:Cell adhesion molecules (CAMs)	4	1.28E−02
	hsa04612:Antigen processing and presentation	3	1.84E−02
	hsa00240:Pyrimidine metabolism	3	2.26E−02
	hsa00230:Purine metabolism	3	4.26E−02
	hsa04060:Cytokine-cytokine receptor interaction	4	4.53E−02

*BPs* biology processes, *KEGG* Kyoto Encyclopedia of Genes and Genomes

### Protein–protein interaction network analysis

**Fig. 3:**
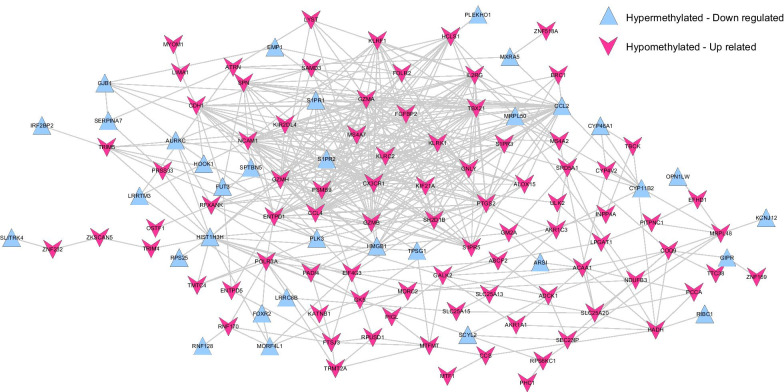
The protein–protein interaction network of genes with opposite directions of expression and methylation levels. The blue triangle represents hypermethylated downregulated genes, the red triangle represents hypomethylated upregulated genes

In the protein–protein interaction network, a total of 119 nodes were identified. This included 33 hypermethylated, downregulated genes and 86 hypomethylated, upregulated genes with 426 pairs of co-expression interactions (Fig. 3).

As shown in Table 2, these genes were significantly associated with 16 GO terms and 10 KEGG pathways. GO functional analysis found that these nodes were primarily involved in functions such as cellular defense response and oxidation reduction. KEGG pathways were primarily involved in natural killer cell mediated cytotoxicity, steroid hormone biosynthesis, and neuroactive ligand-receptor interaction.

**Table 2:** GO BPs and KEGG signal pathways enrichment analysis in interaction network

Category	Term	Count	PValue
Biology process	GO:0006968 ~ cellular defense response	5	9.48E−04
	GO:0006952 ~ defense response	13	1.46E−03
	GO:0055114 ~ oxidation reduction	12	5.95E−03
	GO:0002696 ~ positive regulation of leukocyte activation	5	7.07E−03
	GO:0050867 ~ positive regulation of cell activation	5	8.29E−03
	GO:0051129 ~ negative regulation of cellular component organization	5	1.90E−02
	GO:0016310 ~ phosphorylation	12	2.78E−02
	GO:0042330 ~ taxis	5	2.80E−02
	GO:0006935 ~ chemotaxis	5	2.80E−02
	GO:0002694 ~ regulation of leukocyte activation	5	3.14E−02
	GO:0050865 ~ regulation of cell activation	5	3.71E−02
	GO:0001817 ~ regulation of cytokine production	5	4.11E−02
	GO:0006796 ~ phosphate metabolic process	13	4.51E−02
	GO:0006793 ~ phosphorus metabolic process	13	4.51E−02
	GO:0006468 ~ protein amino acid phosphorylation	10	4.96E−02
	GO:0009617 ~ response to bacterium	5	5.00E−02
KEGG pathway	hsa04650:Natural killer cell mediated cytotoxicity	5	3.35E−03
	hsa00280:Valine, leucine and isoleucine degradation	3	6.19E−03
	hsa00140:Steroid hormone biosynthesis	3	6.69E−03
	hsa04080:Neuroactive ligand-receptor interaction	6	8.55E−03
	hsa00590:Arachidonic acid metabolism	3	9.40E−03
	hsa04612:Antigen processing and presentation	3	1.78E−02
	hsa00240:Pyrimidine metabolism	3	2.19E−02
	hsa04514:Cell adhesion molecules (CAMs)	3	3.46E−02
	hsa00230:Purine metabolism	3	4.16E−02
	hsa04060:Cytokine-cytokine receptor interaction	4	4.39E−02

*BPs* biology processes,* KEGG* Kyoto Encyclopedia of Genes and Genomes

### Analysis of miRNA–target gene network

**Fig. 4:**
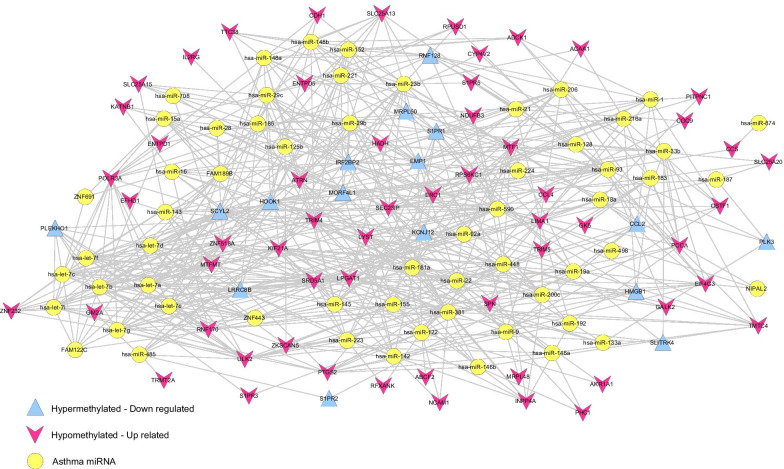
MiRNA regulatory network. The blue triangle represents hypermethylated downregulated genes, the red triangle represents hypomethylated upregulated genes, and the yellow circle indicates miRNAs directly related to asthma

A total of 73 miRNAs that were directly associated with asthma were screened. We screened the target genes of these 73 miRNAs, and then compared the target genes with 130 genes whose expression and methylation level differed significantly. A total of 635 pairs were screened, and the constructed miRNA-mRNA regulatory network contains 133 nodes and 635 connected edges (Fig. 4).

As noted in Table 3, nine KEGG signaling pathways in the regulatory network were screened. These included valine, leucine, and isoleucine degradation (*P* = 2.40e^− 3^), which is annotated to HADH, PCCA, and ACAA1; pyrimidine metabolism (*P* = 9.54e^− 3^), which is annotated to ENTPD5, POLR3A, and ENTPD1; adhesion molecules (*P* = 1.64e^− 2^), which is annotated to NCAM1, CDH1, and SPN; neuroactive ligand-receptor interaction (*P* = 1.65e^− 2^), which is annotated to in S1PR2, S1PR3, S1PR1, and S1PR5; primary immunodeficiency (*P* = 1.76e^− 2^), which is annotated to IL2RG, and RFXANK; fatty acid metabolism (*P* = 1.99e^− 2^), which is annotated to HADH and ACAA1; purine metabolism (*P* = 2.05e^− 2^), which is annotated to ENTPD5, POLR3A, and ENTPD1; cytosolic DNA-sensing pathway (*P* = 2.63e^− 2^), which is annotated to POLR3A and CCL4, and cytokine − cytokine receptor interaction (*P* = 4.27e^− 2^), which is annotated to CCL2, IL2RG, and CCL4.

**Table 3:** The KEGG pathways significantly related to target genes in miRNA regulatory network

Term	Count	PValue	Genes
hsa00280:Valine, leucine and isoleucine degradation	3	2.40E−03	HADH, PCCA, ACAA1
hsa00240:Pyrimidine metabolism	3	9.54E−03	ENTPD5, POLR3A, ENTPD1
hsa04514:Cell adhesion molecules (CAMs)	3	1.64E−02	NCAM1, CDH1, SPN
hsa04080:Neuroactive ligand-receptor interaction	4	1.65E−02	S1PR2, S1PR3, S1PR1, S1PR5
hsa05340:Primary immunodeficiency	2	1.76E−02	IL2RG, RFXANK
hsa00071:Fatty acid metabolism	2	1.99E−02	HADH, ACAA1
hsa00230:Purine metabolism	3	2.05E−02	ENTPD5, POLR3A, ENTPD1
hsa04623:Cytosolic DNA-sensing pathway	2	2.63E−02	POLR3A, CCL4
hsa04060:Cytokine-cytokine receptor interaction	3	4.27E−02	CCL2, IL2RG, CCL4

*KEGG* Kyoto Encyclopedia of Genes and Genomes

### Construction of a pathway network directly related to asthma

**Fig. 5:**
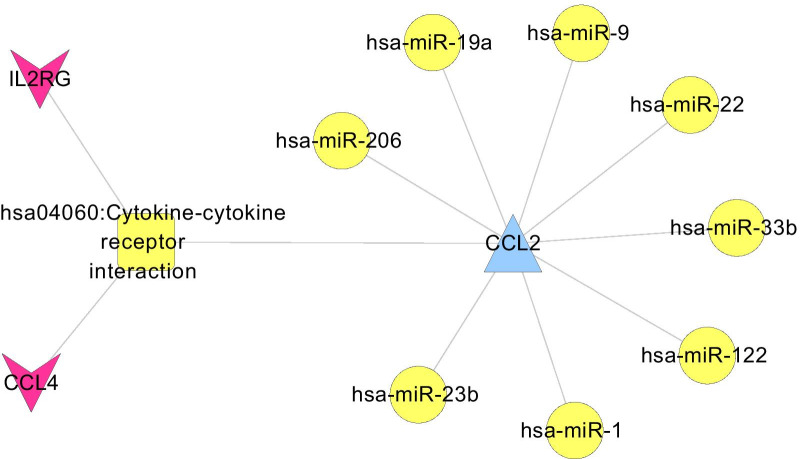
Genes directly related to asthma and KEGG pathways. The blue triangle represents hypermethylated downregulated genes, the red triangle represents hypomethylated up-regulated genes, the yellow circle represents miRNAs directly related to asthma, and the yellow square represents KEGG signal pathways directly related to asthma. KEGG, Kyoto Encyclopedia of Genes and Genomes

We screened 119 KEGG pathways and 116 genes that were directly associated with asthma by searching the CTD database. After comparison with the genes in the constructed regulatory network and the pathways in which genes participate significantly, an overlapping KEGG signaling pathway, hsa04060: cytokine-cytokine receptor interaction, was obtained in which the C−C motif chemokine ligand 2 (*CCL2*) gene directly related to asthma is involved. This gene is targeted by eight asthma related miRNAs (*hsa-miR-206*, *hsa-miR-19a*, *hsa-miR-9*, *hsa-miR-22*, *hsa-miR-33b*, *hsa-miR-122*, *hsa-miR-1*, and *hsa-miR-23b*). As noted in Fig. 5, two additional genes, *IL2RG* and *CCl4*, are involved in this pathway.

## Discussion

Asthma is a complex multifactorial disease caused by the interaction of genetic and environmental factors. In our study, the hub genes were explored via an analysis of multiple data sets that included samples from asthmatics and healthy controls. A total of 130 critical DEGs that were differentially expressed in DNA methylome were detected. In the miRNA−target gene regulatory network directly related to asthma, an overlapping KEGG pathway, hsa04060: cytokine−cytokine receptor interaction, was noted, in which the *CCL2* gene directly related to asthma is involved, and the gene is targeted by 8 asthma related miRNAs. Two other genes, *IL2RG* and *CCL4*, are known to be involved in this pathway.

*CCL2*, also known as MCP-1, is one of several cytokine genes clustered on the q-arm of chromosome 17. Chemokines are a superfamily of secreted proteins involved in immunoregulatory and inflammatory processes. CCL2 is a member of the CC subfamily which is characterized by two adjacent cysteine residues. It binds to chemokine receptors CCR2 and CCR4. The results of our study also show that CCL2 and CCl4 are involved in the cyclokine receptor interaction signaling pathway. CCL2 is closely involved in the inflammatory response in children with asthma [26]. Multiple miRNAs have been shown to regulate the occurrence of inflammation in different diseases through CCL2. Roff et al. reported that microRNA-570-3p regulates HuR and cytokine (CCL2 and CCL4) expression in airway epithelial cells [27]. Downregulation of CCL2 induced by the upregulation of microRNA206 is associated with the severity of HEV71 encephalitis [28]. Chen et al. reported that miR-22 is downregulated in PBMCs from patients with coronary artery disease, and that miR-22 may participate in the inflammatory response by targeting MCP-1 [29]. These findings are consistent with the results of this study.

Interleukin 2 receptor subunit gamma, the protein encoded by *IL2RG*, is an important signaling component of many interleukin receptors, including IL-2, IL-4, IL-7, and IL-21, and is thus referred to as the common gamma chain [30]. Mutations in this gene cause X-linked severe combined immunodeficiency as well as X-linked combined immunodeficiency, a less severe immunodeficiency disorder [31]. The pathway analysis showed that *IL2RG* was involved in primary immunodeficiency and cytokine-cytokine receptor interaction. We speculate that *IL2RG* and *CCL4* might be important genes related to childhood atopic asthma, and together with *CCL2* participate in the cytokine receptor interaction signaling pathway that plays a role in childhood atopic asthma.

There are some limitations in this study. The key genes obtained in this study have not been further verified. In addition, the samples in these two data sets are all PBMCs, which would be more convincing if they were airway epithelial cells. But our research provides new biological insights into the development of asthma.

## Conclusions

In conclusion, our study identified a total of 130 DEGs with significant DNA methylation changes. In a regulatory network directly related to asthma, the KEGG signaling pathway hsa04060:cyclokine-cyclokine receptor interaction was found, in which the *CCL2 *gene, directly related to asthma, is involved, and this gene is targeted by eight asthma related miRNAs. We speculate that *IL2RG* and *CCL4*, which are also involved in this pathway, might be critical genes related to childhood specific asthma, and together with *CCL2* play a role in this disease. The bioinformatics analysis in this study may provide a valuable and reliable basis for determining biomarkers for the development of childhood atopic asthma.

## Data Availability

The datasets that were analysed in this study are available in the NCBI database under the Accession Numbers GSE40736, GSE40732, and GSE40576.

## Abbreviations

DEGs: differentially expressed genes
DMGs: differentially methylated genes
FDR: false discovery rate
GO: gene ontology
HMDD: human MicroRNA disease database
KEGG: Kyoto Encyclopedia of Genes and Genomes

## References

[1] Mukherjee AB, Zhang Z (2011). Allergic asthma: influence of genetic and environmental factors. J Biol Chem.

[2] Asher I, Pearce N (2014). Global burden of asthma among children. Int J Tuberc Lung Dis Off J Int Union Against Tuberc Lung Dis.

[3] Namara B, Nash S, Lule SA, Akurut H, Mpairwe H, Akello F, Tumusiime J, Kizza M, Kabagenyi J, Nkurunungi G (2017). Effects of treating helminths during pregnancy and early childhood on risk of allergy-related outcomes: Follow-up of a randomized controlled trial. Pediatr Allergy Immunol.

[4] Youssef MM, El-Din E, AbuShady MM, El-Baroudy NR, Abd El Hamid TA, Armaneus AF, El Refay AS, Hussein J, Medhat D, Latif YA (2018). Urinary bisphenol A concentrations in relation to asthma in a sample of Egyptian children. Hum Exp Toxicol.

[5] Ober C, Hoffjan S (2006). Asthma genetics 2006: the long and winding road to gene discovery. Genes Immun.

[6] Pinto LA, Stein RT, Kabesch M (2008). Impact of genetics in childhood asthma. J Pediatr.

[7] Nakashima K, Hirota T, Suzuki Y, Akahoshi M, Shimizu M, Jodo A, Doi S, Fujita K, Ebisawa M, Yoshihara S (2006). Association of the RIP2 gene with childhood atopic asthma. Allergol Int Off J Jpn Soc Allergol.

[8] Qian XB, Wu Y, Cao SY, Cai XH, Yu CY, Xuan MY, Cao SS, Li XC (2011). Association of single nucleotide polymorphisms in the promoter region of the TLR9 gene with childhood atopic asthma. Chin J Med Genet..

[9] Stefanowicz D, Hackett TL, Garmaroudi FS, Günther OP, Neumann S, Sutanto EN, Ling KM, Kobor MS, Kicic A, Stick SM (2012). DNA methylation profiles of airway epithelial cells and PBMCs from healthy, atopic and asthmatic children. PLoS ONE.

[10] Acevedo N, Reinius LE, Greco D, Gref A, Orsmark-Pietras C, Persson H, Pershagen G, Hedlin G, Melén E, Scheynius A (2015). Risk of childhood asthma is associated with CpG-site polymorphisms, regional DNA methylation and mRNA levels at the GSDMB/ORMDL3 locus. Hum Mol Genet..

[11] Nicodemus-Johnson J, Naughton KA, Sudi J, Hogarth K, Naurekas ET, Nicolae DL, Sperling AI, Solway J, White SR, Ober C (2016). Genome-wide methylation study identifies an IL-13-induced epigenetic signature in asthmatic airways. Am J Respir Crit Care Med.

[12] Brand S, Kesper DA, Teich R, Kilic-Niebergall E, Pinkenburg O, Bothur E, Lohoff M, Garn H, Pfefferle PI, Renz H (2012). DNA methylation of TH1/TH2 cytokine genes affects sensitization and progress of experimental asthma. J Allergy Clin Immunol.

[13] Yang IV, Pedersen BS, Liu A, O’Connor GT, Teach SJ, Kattan M, Misiak RT, Gruchalla R, Steinbach SF, Szefler SJ (2015). DNA methylation and childhood asthma in the inner city. J Allergy Clin Immunol.

[14] Barrett T, Wilhite SE, Ledoux P, Evangelista C, Kim IF, Tomashevsky M, Marshall KA, Phillippy KH, Sherman PM, Holko M (2013). NCBI GEO: archive for functional genomics data sets–update. Nucleic Acids Res.

[15] Ritchie ME, Phipson B, Wu D, Hu Y, Law CW, Shi W, Smyth GK (2015). limma powers differential expression analyses for RNA-sequencing and microarray studies. Nucleic Acids Res.

[16] Wang L, Cao C, Ma Q, Zeng Q, Wang H, Cheng Z, Zhu G, Qi J, Ma H, Nian H (2014). RNA-seq analyses of multiple meristems of soybean: novel and alternative transcripts, evolutionary and functional implications. BMC Plant Biol.

[17] Szekely GJ, Rizzo ML (2005). Hierarchical clustering via joint between-within distances: extending Ward’s minimum variance method. J Classif.

[18] Press WT, Vetterling SA, Flannery WT. BP: Sec. 16.4. Hierarchical clustering by phylogenetic trees. In; 2007.

[19] Huang da W, Sherman BT, Lempicki RA (2009). Systematic and integrative analysis of large gene lists using DAVID bioinformatics resources. Nat Protoc.

[20] Huang da W, Sherman BT, Lempicki RA (2009). Bioinformatics enrichment tools: paths toward the comprehensive functional analysis of large gene lists. Nucleic Acids Res.

[21] Szklarczyk D, Morris JH, Cook H, Kuhn M, Wyder S, Simonovic M, Santos A, Doncheva NT, Roth A, Bork P (2017). The STRING database in 2017: quality-controlled protein–protein association networks, made broadly accessible. Nucleic Acids Res.

[22] Shannon P, Markiel A, Ozier O, Baliga NS, Wang JT, Ramage D, Amin N, Schwikowski B, Ideker T (2003). Cytoscape: a software environment for integrated models of biomolecular interaction networks. Genome Res.

[23] Huang Z, Shi J, Gao Y, Cui C, Zhang S, Li J, Zhou Y, Cui Q (2019). HMDD v3.0: a database for experimentally supported human microRNA-disease associations. Nucleic Acids Res.

[24] Li JH, Liu S, Zhou H, Qu LH, Yang JH (2014). starBase v2.0: decoding miRNA–ceRNA, miRNA–ncRNA and protein–RNA interaction networks from large-scale CLIP-Seq data. Nucleic Acids Res.

[25] Davis AP, Grondin CJ, Johnson RJ, Sciaky D, McMorran R, Wiegers J, Wiegers TC, Mattingly CJ (2019). The comparative toxicogenomics database: update 2019. Nucleic Acids Res.

[26] Lewis TC, Henderson TA, Carpenter AR, Ramirez IA, McHenry CL, Goldsmith AM, Ren X, Mentz GB, Mukherjee B, Robins TG (2012). Nasal cytokine responses to natural colds in asthmatic children. Clin Exp Allergy J Br Soc Allergy Clin Immunol.

[27] Roff AN, Craig TJ, August A, Stellato C, Ishmael FT (2014). MicroRNA-570-3p regulates HuR and cytokine expression in airway epithelial cells. Am J Clin Exp Immunol.

[28] Zhang G, Wang J, Yao G, Shi B (2017). Downregulation of CCL2 induced by the upregulation of microRNA-206 is associated with the severity of HEV71 encephalitis. Mol Med Rep.

[29] Chen B, Luo L, Zhu W, Wei X, Li S, Huang Y, Liu M, Lin X (2016). miR-22 contributes to the pathogenesis of patients with coronary artery disease by targeting MCP-1: an observational study. Medicine.

[30] Impellizzieri D, Ridder F, Raeber ME, Egholm C, Woytschak J, Kolios AGA, Legler DF, Boyman O (2019). IL-4 receptor engagement in human neutrophils impairs their migration and extracellular trap formation. J Allergy Clin Immunol.

[31] Lim CK, Abolhassani H, Appelberg SK, Sundin M, Hammarström L (2019). IL2RG hypomorphic mutation: identification of a novel pathogenic mutation in exon 8 and a review of the literature. Allergy Asthma Clin Immunol Off J Can Soc Allergy Clin Immunol..

